# Osteocondritis dissecans lesions of the knee restored by bone marrow aspirate concentrate. Clinical and imaging results in 18 patients

**DOI:** 10.1007/s00590-022-03214-1

**Published:** 2022-02-08

**Authors:** Matteo Baldassarri, Roberto Buda, Luca Perazzo, Diego Ghinelli, Ricciardello Sarino, Brunella Grigolo, Cesare Faldini

**Affiliations:** 1 Villa Maria Hospital, Viale Matteotti, 4 - 47921 Rimini, Italy; 2 Orthopaedic and Traumatology Clinic, Ospedale “SS. Annunziata” - Università degli Studi Gabriele d’Annunzio - Chieti e Pescara, Via dei Vestini, 31 - 66100 Chieti, Italy; 3 Casa di Cura Villa Laura, Via Emilia Levante, 137 - 40139 Bologna, Italy; 4 RAMSES Laboratory, Rizzoli Orthopaedic Institute, Via Giulio Cesare Pupilli 1 - 40136, Bologna, Italy; 5 I Orthopaedic and Traumatology Clinic, Rizzoli Orthopaedic Institute – Bologna, Viale G. C. Pupilli, 1 - 40100 Bologna, Italy; 6 UNIZKM: Università Cattolica Nostra Signora del Buon Consiglio, Tirana – Albania, Via A. Poliziano, 2 - 40129 Bologna (BO), Italy

**Keywords:** Osteochondritis dissecans (OCD), Cartilage, Knee, Bone marrow-derived cell transplantation (BMDCT)

## Abstract

**Background:**

Osteochondritis dissecans (OCD) is a common cartilage disorder that specifically affects the knees of skeletally immature and young adult patients. There have been a few treatments that have been proposed: fixation of the fragment, drilling, microfractures. The aim of this study was to analyze retrospectively clinical and imaging results obtained by treating it with one-step bone marrow-derived cells Transplantation (BMDCT) technique.

**Methods:**

From 2007 to 2014, 18 patients (mean-age 19.1 ± 5.0 years) affected by OCD were treated with one-step BMDC transplantation. In our observational study, clinical evaluation was performed at a scheduled follow-up through IKDC, Tegner, KOOS and EQ-VAS. X-rays and MRI were conducted preoperatively and at 12 months. At final follow-up, MRI MOCART Score was evaluated.

**Results:**

IKDC and KOOS clinical scores showed a progressive increase. Tegner Score at final follow-up (5.3 ± 2.7) was significantly lower compared to the pre-injury level (6.5 ± 2.1); however, these results showed a statistically significant improvement that remained over time. EQ-VAS showed a significant improvement in every follow-up measure. MRI Mocart Score showed a complete or almost complete filling of the lesion in 13 patients.

**Conclusions:**

“One-step” technique allows articular surface restoration with viable physiologic osteochondral tissue with a high clinical efficacy and imaging results. The number of cases is still limited, and further studies with larger sample sizes and greater follow-up evaluations are required to confirm our results. Nevertheless, we believe that BMDCT may represent a suitable option to treat OCD lesion in young adults.

## Introduction

Osteochondritis dissecans (OCD) is a common cartilage disorder that affects specially the knee causing pain and dysfunction among skeletally immature and young adult patients. Formerly in 1870, a French surgeon Ambroise Paré was the first to identify a fragment of cartilage (loose body) in the knee in a young man. It was König in 1888 that coined the definition as well-known today [1–3].

OCD is an acquired, idiopathic lesion of subchondral bone, potentially reversible, which can lead to secondary instability in the overlaying cartilage. OCD is postulated to primarily stem from repetitive microtrauma, ischaemia factors, genetic influences, and growth disturbances may also play a role [4]. Its incidence mostly affects young patients between 10 and 20 years of age, with a prevalence of 15–29 per 100,000 population [5, 6]. The most common locations of OCD of the knee are the medial femoral condyle (70–80%), lateral femoral condyle (15–20%), and patella (5–10%) [7]. Bilateral OCD of the knee has been reported in 12–30% of cases [8]. When an adult was involved, OCD is thought to be owing to persistence of an unresolved juvenile OCD lesion (JOCD), although de novo adult OCD lesions have been reported and both adult and juvenile OCD lesions have potential for later sequelae (particularly premature degenerative joint disease) [9].

Patients with a suspected OCD of the knee should be evaluated with radiographs including AP, lateral, sunrise/merchant, and tunnel views, but a magnetic resonance imaging (MRI) is most commonly used to further evaluate the lesion. MRI allows characterization of the lesion as stable or unstable [10]. Many researchers propose treatment with different approaches: currently, it is well established that nonoperative early management is indicated for stable lesions in skeletally immature patients [11, 12]. Conversely, surgical treatment is recommended for any detached or unstable lesions in patients nearing physeal closure or when the complaints are severe (intense pain, effusion, and generalised weakness). Surgical options include debridement, drilling, microfracture, reduction and fixation, autograft osteochondral transplantation, autologous chondrocyte implantation, and allograft osteochondral transplantation [13–20].

In the last decade, a new bone marrow-derived cells (BMDC) so-called “One-step technique” technique has been developed for the treatment of osteochondral defects obtaining several successes and achievements in the treatment of focal cartilage lesions of the knee and ankle [21, 22]. On the wave of such promising results, we decided to treat lesions with bone marrow-derived cells transplantation (BMDCT) technique.

Therefore, the aim of this study was to analyse clinical and imaging results of the BMDCT one-step technique, from a sample of 18 individuals.

## Materials and methods

From June 2007 to August 2014, 18 patients (11 males and 7 females with mean-age of 19.1 ± 5.0 years) affected by OCD of the femoral condyles were treated with one-step BMDC transplantation. Inclusion criteria consisted of patients between 15 and 30 years who complained of typical OCD symptoms such as knee pain, swelling, and locking and then failed 3–6 months of conservative therapies; type II and III lesions according to ICRS classification were included. (Mean lesion size was 2.03 ± 0.6 cm^2^, and mean deep was 7.7 ± 3.9mm.) Exclusion criteria were: advanced osteoarthritis, concomitant anterior cruciate ligament (ACL) or posterior cruciate ligament (PCL) deficiency. Patients with infective, metabolic or inflammatory pathologies were also excluded from the study.

**Fig. 1:**
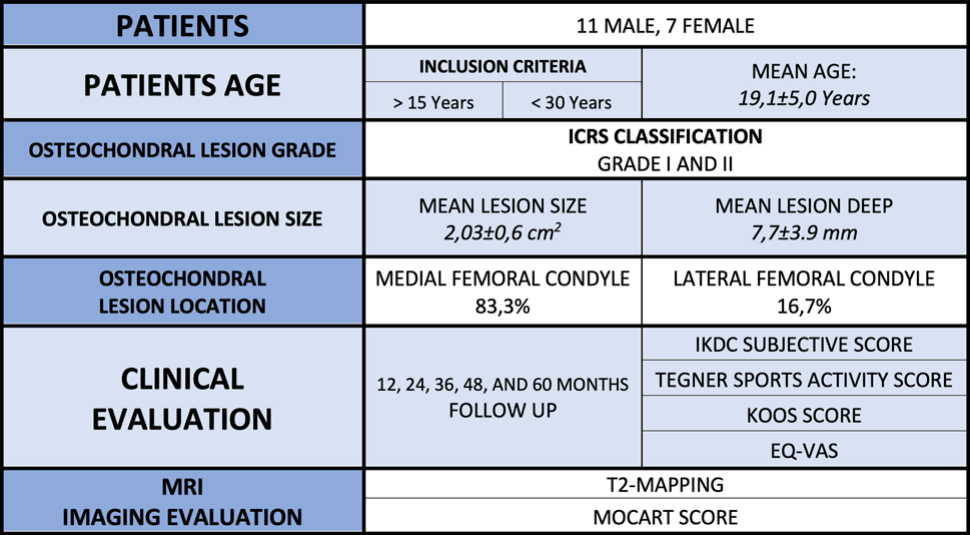
Study summary

The clinical study protocol was approved by an independent Ethical Committee, and signed informed consent for participation to the study was obtained from all the included patients (Fig. 1).

## Surgical technique

All the patients underwent BMDCT through the “one-step” arthroscopic procedure with different phases:

### Platelet gel production

120ml of the patient’s venous blood is harvested and processed through a plasma plateletpheresis procedure, using the Vivostat System (Vivostat, Allerød, Denmark), which allows to obtain 6ml of platelet-rich fibrin gel ready for use.

The gel can be produced just before or in the days preceding the surgery. In this case (the latter), it needs to be frozen and kept at −35°C until the day of the surgery, when it is defrosted slowly, about 30 min before its uses.

The platelet-rich fibrin (PRF) gel is preferred to the platelet-rich plasma (PRP) gel because of its higher fibrin concentration, which allows a faster gelification of the product in the lesion site.

### Bone marrow harvesting

For this surgical procedure, a IOR-G1 Kit (Novagenit S.r.l, Mezzolombardo, TN, Italy) is used. A specific marrow needle (size 11 G9 100 mm) was inserted 3 cm deep into the spongy bone of the posterior iliac crest. The purpose of this step was to fill a 20 ml syringe (internally coated with calcium–heparin solution) with 5ml of bone marrow. Other 5 ml were extracted after rotating by 90 degrees the needle in order to collect 5 ml of bone marrow at each aspiration. In some cases, other rotations were performed with a moderate withdrawing from different points allow to maximize harvesting and reduce blood dilution. The procedure was repeated 3–4 times using the same skin opening. At the end of this procedures, 60ml of bone marrow was obtained.

### Bone marrow concentration

The collected bone marrow is processed through a centrifuge (Res-Q, Thermogenesis, Rancho Cordova, CA) directly in the operating room which allows to remove most of the erythrocytes and plasma and to obtain 10 ml of concentrate, containing the total nuclear and mononuclear fractions (TNC and MNC). The system is made of a 60 mL centrifuge tube to collect the bone marrow aspirate. The device is equipped with a special density controlled floating funnel that throughout centrifugation at 3200 rpm for 12 min. It collects a range of 6–10 mL of BMDC containing the buffy coat rich in either nuclear/mononuclear cells or PRP (Platelet-Rich Plasma) and isolating it from erythrocytes and PPP (Platelet Poor Plasm). The device is then placed on the magnetic processing tray that resuspend the buffycoat for the final extraction. The BMC containing TNC/MNC is recovered in a syringe through a recovery tubing and a Luer Lock port in sterile conditions. In conclusion, the system maximizes the percentage yield of total nucleated cells (TNC) and mononuclear cells (MNC), and/or platelets at the point of care, while reducing sample volume by up to 20× [23, 24].

### Arthroscopic transplantation of BMDC

**Fig. 2:**
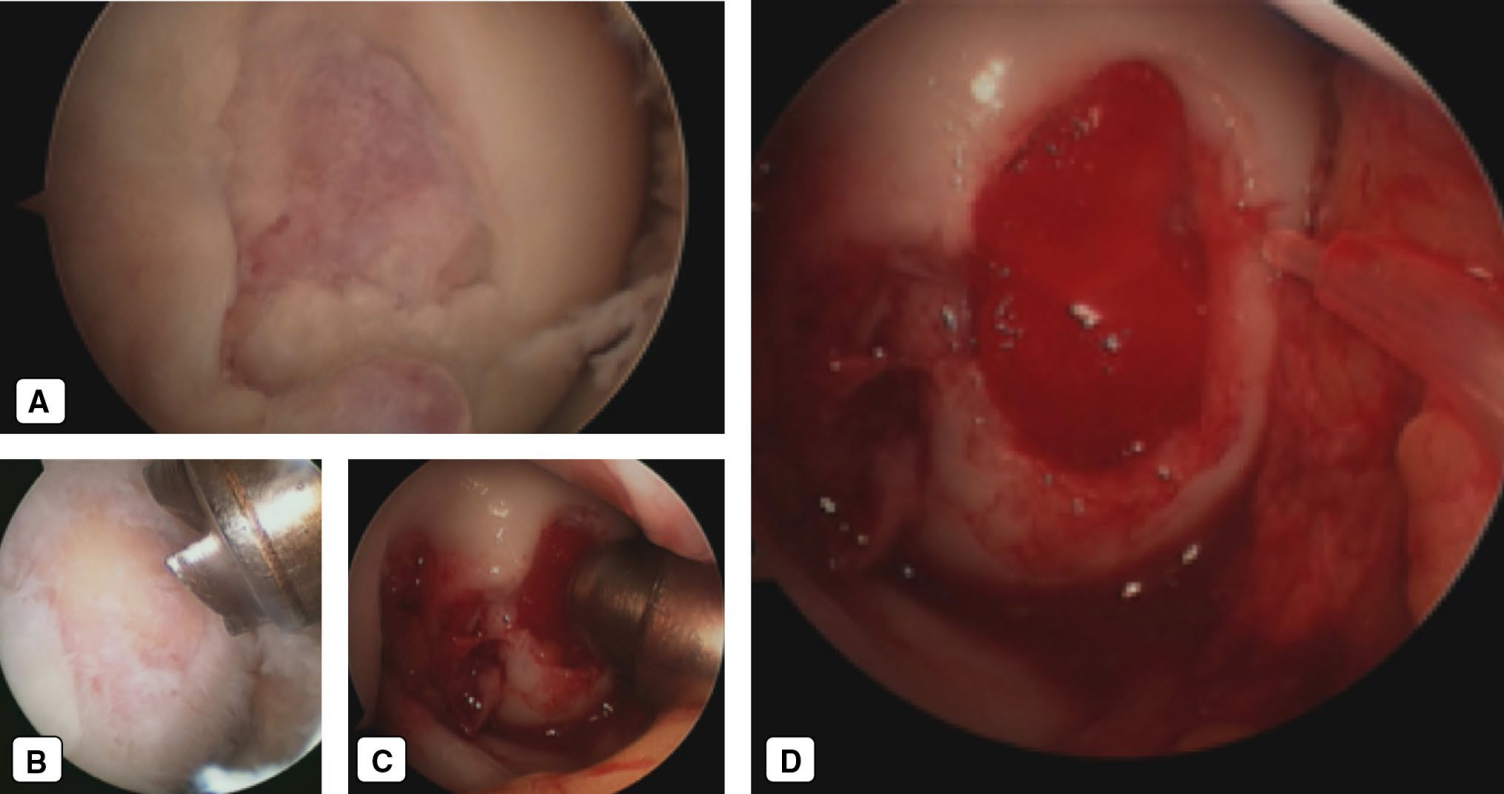
Intraoperative procedure. **a** Osteochondritis dissecans (OCD) lesion—Arthroscopic View. **b** Osteochondritis dissecans (OCD) lesion exposed. **c** Bone marrow-derived cells with scaffold loading in the lesion. **d** Platelet-rich fibrin (PRF) gel spraying on the scaffold

Once the harvesting phase has been completed, the patient is turned to the supine position and a standard knee arthroscopy is initiated. Using antero-medial and antero-lateral standard arthroscopic accesses, the OCD lesion is located and evaluated (Fig. 2a). At this point, damaged cartilage is removed, and the subchondral bone is exposed (Fig. 2b). The depth and surface of the lesion are measured, and a piece of collagen scaffold (Biopad, Novagenit S.r.l, Mezzolombardo, TN, Italy) is cut to fit its size. The sized scaffold is then loaded with 10 mL of bone marrow concentrate and brought inside the joint through an open cannula (Fig. 2c). The concentrate is uniformly spread over the porous collagen-based scaffold. The hydrophilicity and the porosity of the construct allow the fast absorption of BMC and related TNC/MNC fractions. The TNC and MNC significantly increase in a range of 5.8–6.7-fold in comparison to unprocessed bone marrow aspirate. The estimated number of TNC in the BMC is roughly 1.82 × 106 cells in 7 mL, while the number of MNC is 364 × 106.

The biomaterial was regularized using a flat probe, and the previously provided PRF was applied to cover the lesions using a special spray pen (Fig. 2d). PRF is crucial to obtain growth factors and a fibrin clot to fasten the implanted biomaterial and stimulate regeneration. The stability of the implanted scaffold is tested through multiple flexions and extensions of the knee. Conventional closure of the skin was performed.

## Clinical and imaging evaluation

Clinical evaluation with IKDC subjective score, Tegner sports activity score, KOOS score and with EQ-VAS was performed at 12, 24, 36, 48, and at final follow-up of 60 months.

X-rays and high-resolution magnetic resonance imaging (MRI) evaluation were conducted preoperatively in order to assess the characteristics of the lesion, i.e. location, size, ICRS-OCD grade and subchondral oedema. MRI control was also performed at 12 months and at last follow-up.

The medial femoral condyle was involved in 15 (83.3%) patients, whereas the lateral femoral condyle was affected only in 3 (16.7%) patients. In 10 cases, the affected knee was the right one, in 8 the left one.

## Post-operative rehabilitation

Continuous passive motion (CPM) (0°–35°; 1 cycle/min) was initiated on the first post-operative day for 6–8 h per day. The literature widely describes beneficial results of CPM enhancing joint nutrition, cartilage healing and lowering rate of post-operative adhesions [24, 25]. Weight bearing must be avoided for the subsequent 6 weeks.

Four weeks after surgery the treatment are intensified with the introduction of muscular reinforcement exercises, closed kinetic-chain and proprioceptive rehabilitation, static exercises and swimming.

Six weeks after surgery partial and gradual weight-bearing are allowed, and extension exercises are introduced.

Open kinetic-chain exercises and cycling, together with complete weight-bearing, are introduced only 10 weeks after surgery. In-line running is allowed 6 months after surgery, whereas high-impact sports only after 12 months.

## Magnetic resonance imaging

All the MRI studies available, performed before and after surgical treatment, were evaluated. MRI has been performed in several medical centre with different MRI equipments. The studies included only MRI's performed with at least 1.5 Tesla Magnet and acquired with standard protocols (T1 coronal spin echo sequence, sagittal proton density with fat saturation (PD-FS) sequence, axial dual echo with PD-FS and T2 with fat saturation sequence and 3-dimensional PD sequence.

The post-operative MRI-scan at final follow-up was evaluated using the MOCART (Magnetic Resonance Observation of Cartilage Repair Tissue) Score with additional assessments of subchondral bone marrow and cysts, which allowed a quantitative and morphological assessment of the regenerative tissue, defining both the depth (expressed in mm) and the surface (expressed in mm^2^) of the new tissue.

MRI scans were scored in a blinded method by a musculoskeletal radiologist, with MOCART score (score of 0 [worst] to 100 [best]).

### Statistical analysis

The clinical and radiological results were statistically correlated. All continuous variables were expressed in terms of mean and standard deviation. Specific analysis is made to evaluate significant differences of the clinical score trend along the follow-up. For repeated measures, the ANOVA test was used. For multiple comparisons for repeated measures, Sidak correction is used. To assess the influence on clinical scores of the variables, the Mann–Whitney for small samples was used. To assess the influence of continuous or rank variables on clinical scores, the Spearman rank correlation test was used.

For all the tests, a *p* value <0.05 was considered significant. The analysis was performed using the Statistical Package for the Social Sciences (SPSS) software version 15.0 (SPSS Inc., Chicago, the USA).

## Results

### Clinical results

**Fig. 3:**
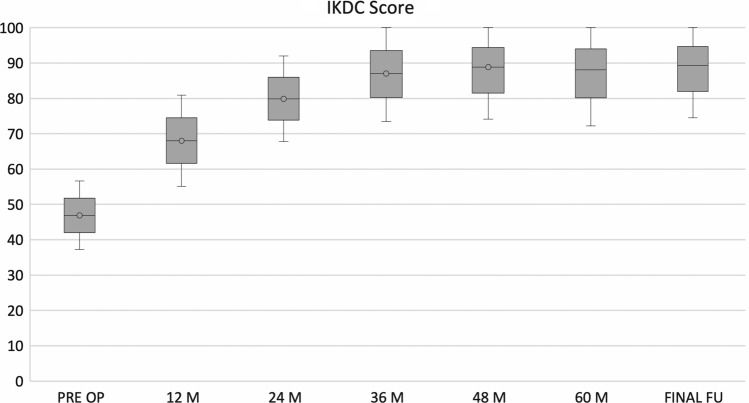
IKDC subjective score

**Fig. 4:**
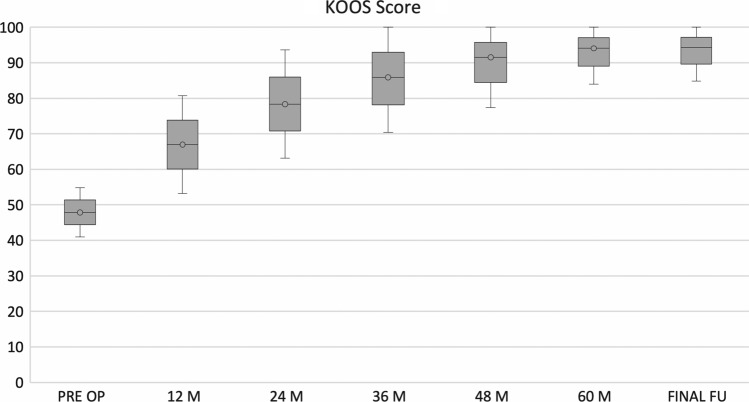
Knee Injury and Osteoarthritis Outcome Score (KOOS)

**Fig. 5:**
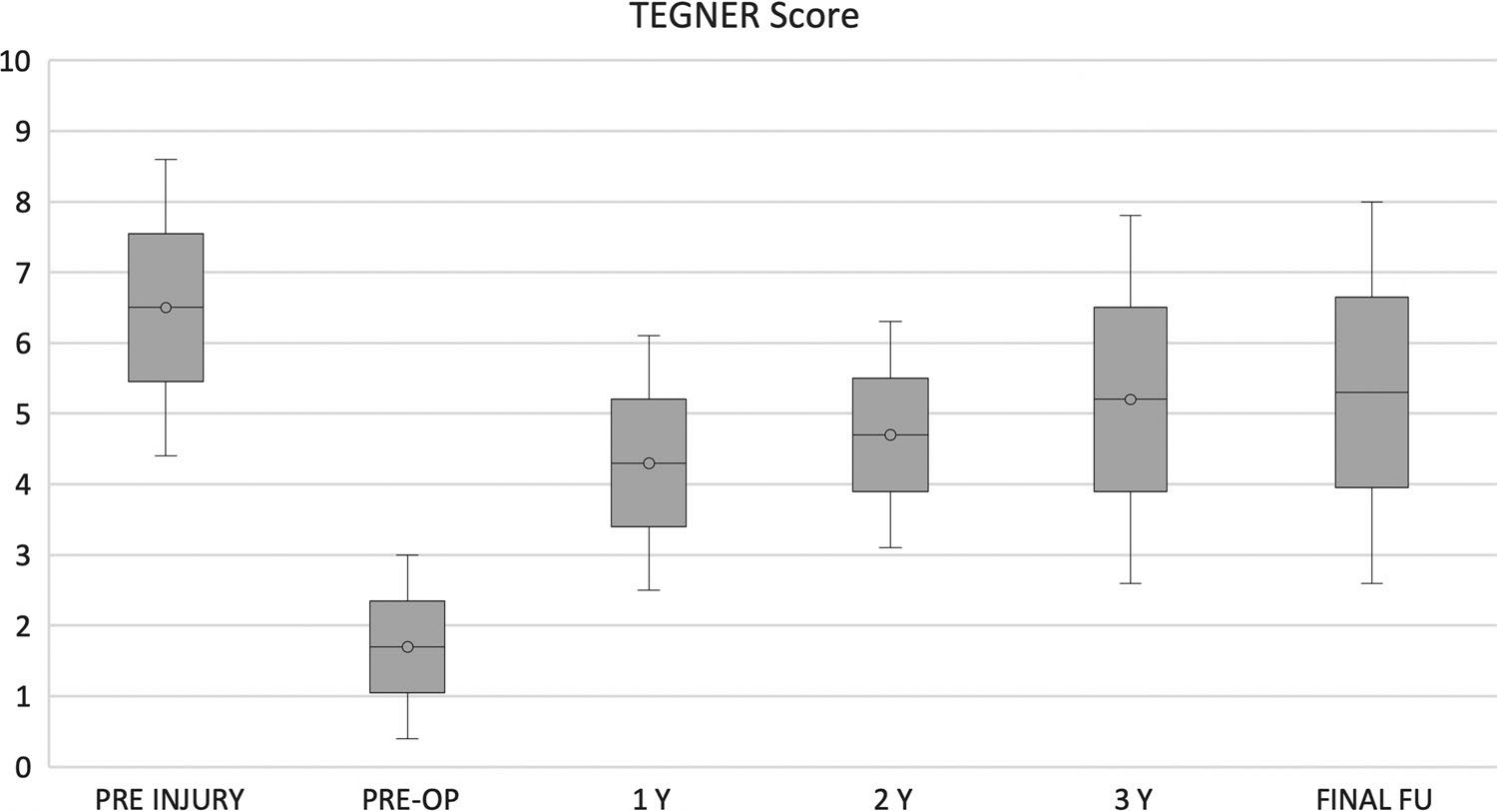
TEGNER Sports Activity Score

No post-operative complications were observed. IKDC and KOOS clinical scores showed a progressive increase from pre-surgery level confirmed at the last follow-up. In particular, mean IKDC subjective score at final follow-up was 89.3 ± 8.1 (*p *< 0.005) compared to the pre-operative score of 46.8 ± 9.7. Analysing the collected data, the IKDC score results at the designated follow-ups had statistically significant increases (Fig. 3). The KOOS score mean value at final follow-up was 94.3 ± 9.4 (*p* < 0.005) compared to pre-operative score of 47.8 ± 6.8. In particular as for IKDC score, KOOS score was improved, with statistically significance, at every established follow-up (Fig. 4). The pre-injury Tegner score was 6.5 ± 2.1, while the pre-operative value was 1.7 ± 1.3. This improved to 5.2 ± 2.6 at 3-year follow-up and to 5.3 ± 2.7 at the final follow-up. The level of sport activity at 2 and 3 years was significantly lower compared to the pre-injury level (*p* < 0.05); however, these results showed a statistically significant improvement (*p* < 0.05) stable over time (Fig. 5).

**Fig. 6:**
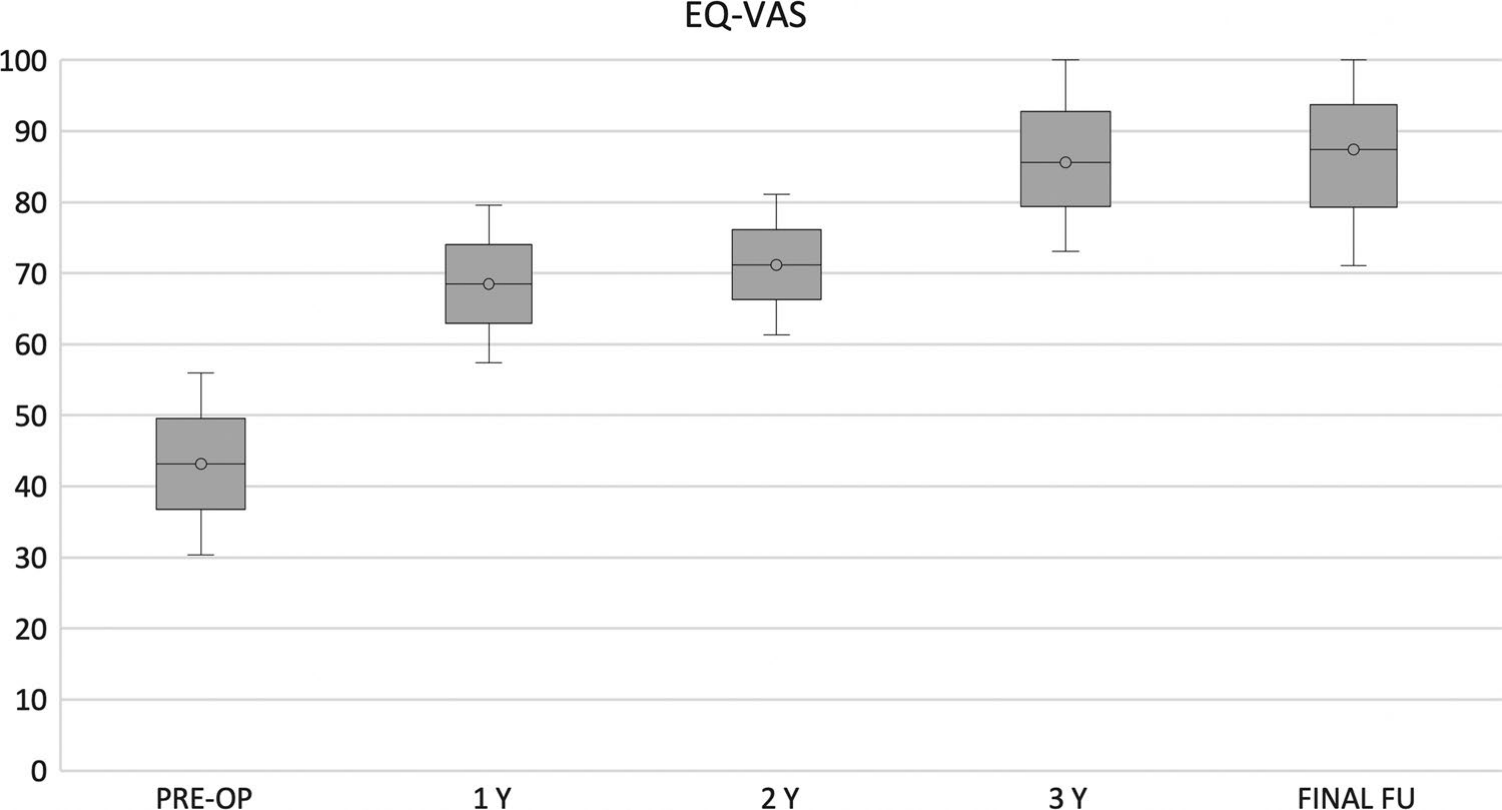
EQ-VAS Score

Self-assessment of quality of life (EQ-VAS) showed a significant improvement from pre-operative evaluation (43 ± 6.8) at 1-year (68.5 ± 5.1) and final follow-ups (87.4 ± 7.3) (*p* < 0.05); (Fig. 6) Regarding patient’s gender, it was noted that both the IKDC and the KOOS pre-op were correlated as statically significant (*p* = 0.001 for the IKDC and *p* = 0.012 for the KOOS) The male subjects tended to start from lower clinical scores than women. However, it was found, although not statistically significant, that at the final follow-up male patients reached higher scores than women.

Analysing lesion’s characteristics, a statistically significant correlation can be observed between the course of the IKDC at 12 months and pre-operative depth of the lesion (*p* = 0.046): the patients who have a lesion’s depth ≤4mm show an IKDC score at 12 months greater than those with a lesion >4 mm deep. This significant result difference disappears in subsequent follow-ups. Lesion’s depth also affects the IKDC pre-op, which is significantly lower (*p* = 0.001) in patients for depth >4mm; similarly, the size of the lesion significantly influences the IKDC pre-op (*p* = 0.013), which is higher in patients with lesions ≤150 mm^2^. No statistically significant correlation between patient’s age and BMI at time of surgery was found.

### MRI results

All the patients recruited underwent the post-operative MRI assessment at 12 and last follow-up. The “DPFSE fat-saturated” MRI images were evaluated using the 3D-MOCART Score.

In 13 patients, a complete (100%) or almost complete (75–100%) filling of the lesion was observed, whereas in 3 patients, the regenerative tissue proved to be hypertrophic. Only 2 patients showed incomplete (<75%) filling of the defect.

The chondral integration of the regenerative tissue with the native hyaline cartilage was complete or almost complete in 14 out of 18 patients. Three of four remaining patients showed a visible defect <50%, 1 patient >50%. 8 patients showed complete osteointegration, proven by the absence of demarcating borders between the regenerative tissue and the surrounding native tissue. The remaining 10 patients showed a good osteointegration, even though signs of partial delamination were still detectable.

The surface of the regenerative tissue proved to be intact in 8 patients, whereas in 9 patients, it was possible to detect some irregularities affecting <50% of the overall thickness of the regenerate. Only 1 patient shows >50% damages of overall thickness. 11 patients showed nearly normal signal intensity in the regenerative tissue; in 3 patients, the signal intensity was abnormal. Six patients present perfectly homogeneous structure of the regenerate tissue, and 7 was inhomogeneous. 5 patients were possible to observe clefts.

The subchondral bone plate was intact or nearly intact (>50%) in 10 patients, whereas abnormalities were shown in 8 patients. The subarticular spongiosa was normal in 10 patients. No adhesion or osteophytes were detected, whereas a small amount of intraarticular effusion was shown in 9 patients. A small area of subchondral oedema could be detected in 8 patients.

When the degree of filling of osteochondral defect resulted complete, this tended to positively impact the clinical scores.

**Fig. 7:**
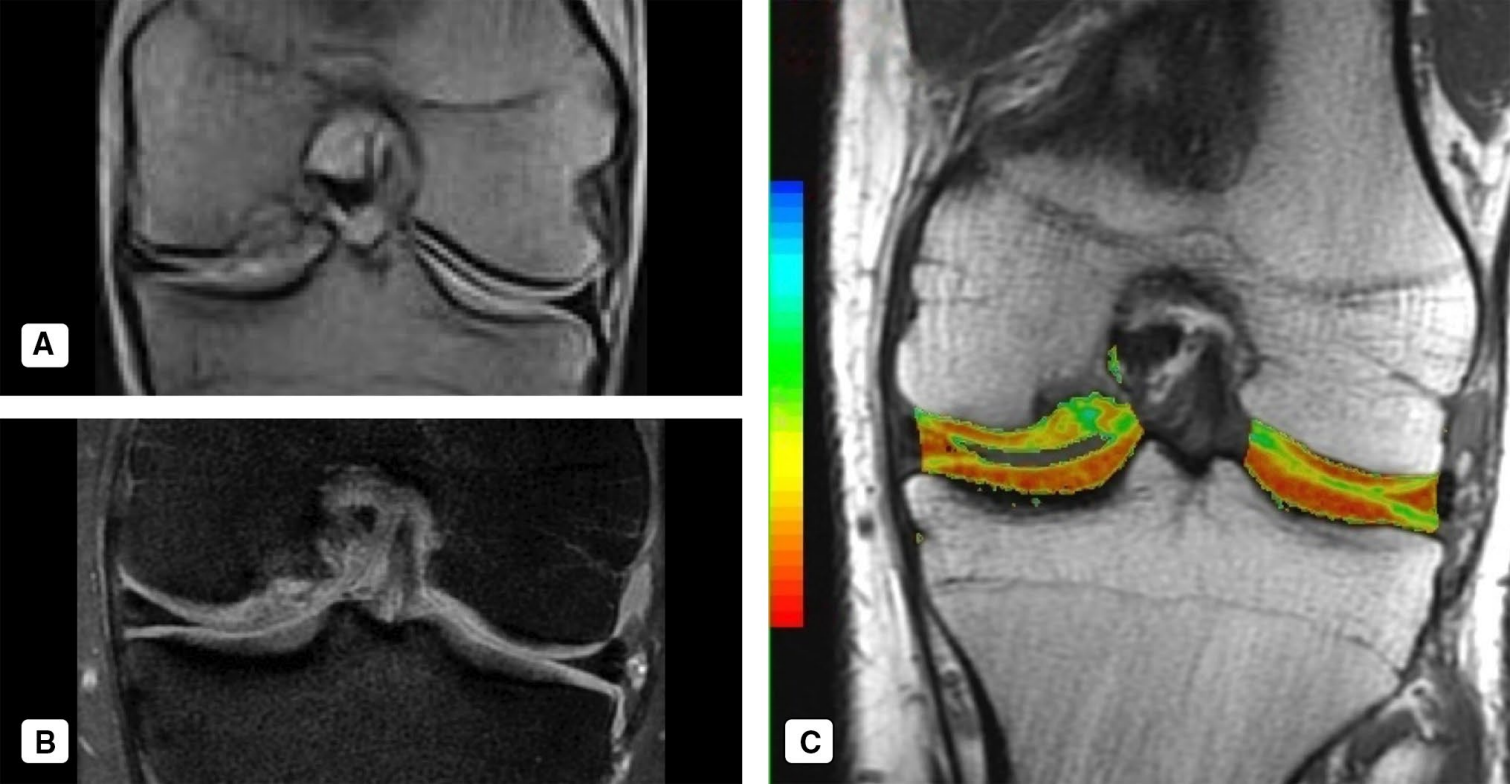
Case imaging. **a** Pre-Op MRI, **b** 12 months follow-up MRI, **c** MOCART Score Evaluation on 12 months follow-up MRI

**Fig. 8:**
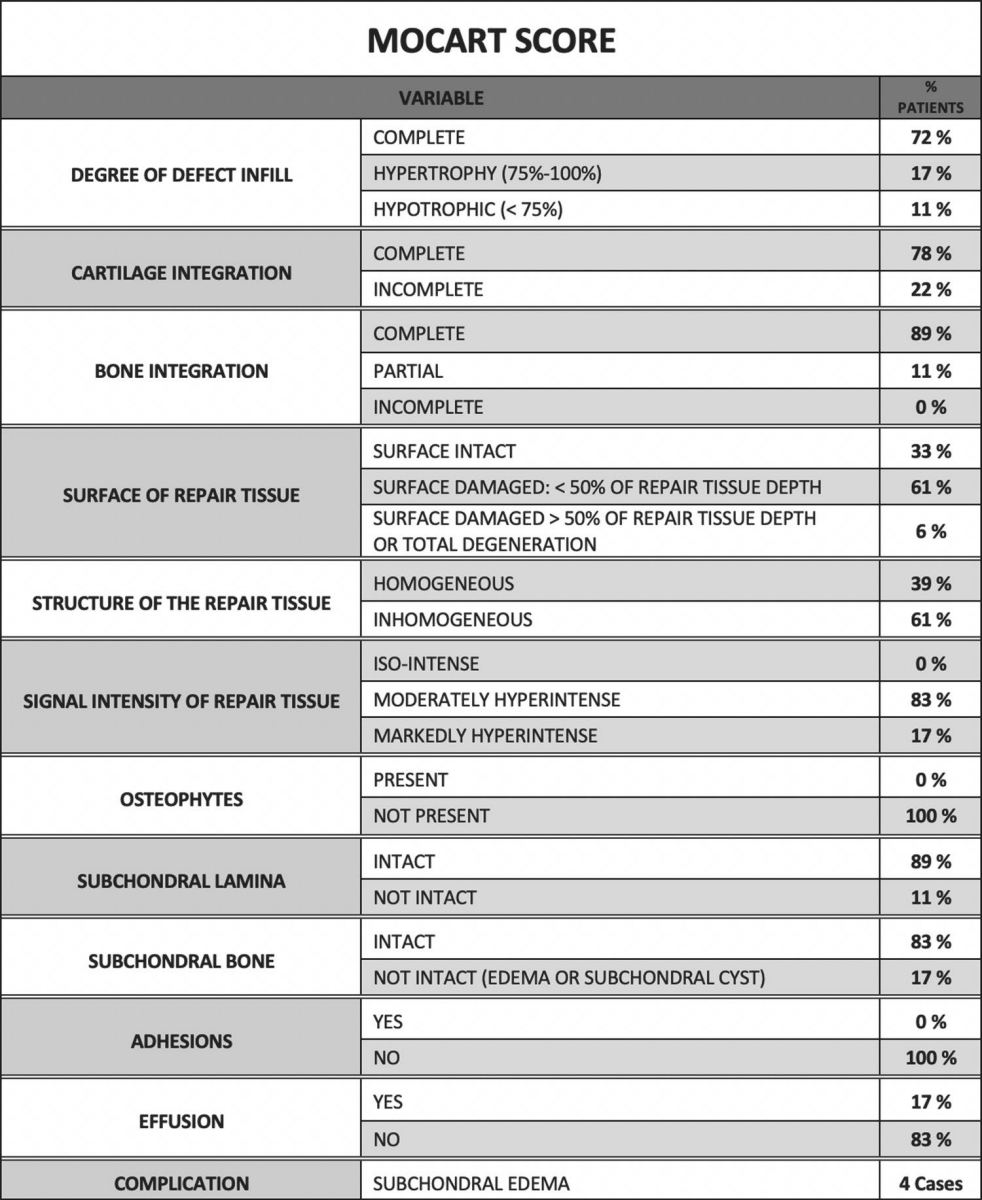
MOCART score at final follow-up

Furthermore, comparing pre-op and final follow-up MRI subchondral bone regeneration was evident in all cases. No correlations were seen between the remaining MOCART parameters and the clinical score (Figs. 7, 8).

## Discussion

OCD today continues to be a pathological entity characterized by a non-unique interpretation of aetiology and histopathological appearance. The main finding of the present study is that the use of BMDC transplantation can mitigate clinical symptoms and function impairment in young patients affected by OCD of the knee, with sustainable results observed at 72 months follow-up. Otherwise, there is much confusion regarding classification and definition of OCD lesions and their differentiation from others, as well as with regard to a clear definition of juvenile OCD and adult OCD. These diagnostic uncertainties often have generated no clear scientifically significant recommendations and consequently raise doubts about the most appropriate surgical strategy, especially among so-called regenerative techniques.

As indicated in the Detterline therapeutic algorithm, fixation of the detached osteochondral fragment is still considered the first-line treatment for osteochondritis dissecans [26]. This option, in fact, is able to restore the native architecture of the osteocartilaginous layer, thereby obtaining a stable and a long-lasting efficacious clinical outcome. However, often the osteochondral fragment cannot be reattached, or it fails to heal after initial fixation [27–29].

Unfortunately, no specific trauma emerged in our case series which led to a striking clinical symptomatology often remains clinically silent in the initial stages. In fact, due to a slow onset of the pathology, from the etiological point of view, the mechanism of microtraumatism had prevailed in our case series. This pathogenesis renders the fixation of the fragment unworkable [30, 31].

Several techniques have been proposed over time in OCD developed to repair chondral surface and adapted to reconstruct the osteochondral damaged area. Brittberg first, in osteochondral defects with ACI technique, proposed to regenerate cartilaginous tissue with biomechanical properties comparable to those of the surrounding healthy cartilage [32]. The procedure showed promising findings also for OCD lesions: some authors in their case series have reported a 37% reoperation rate and 22% failure rate in this setting; furthermore, in other case series, a persistent subchondral oedema-like signals and incomplete maturation of the bone graft at follow-up were mentioned [33].

Another level-IV study reported similar results in 40 exclusively JOCD patients. A follow-up in 80% after the classic ACI treated patients a success rate of 85% was found, while the failure rate was 19% [34] Ferruzzi et al. compared ACI via an arthrotomy (*n* = 48) with an arthroscopic procedure (*n* = 50) using a cell-seeded matrix. They observed a significant improvement in both groups, but the failure rate after an open procedure was 19%, distinctly higher than after the arthroscopic technique (4%). In addition, they noted a faster rehabilitation following arthroscopy-mediated treatment [35].

Otherwise, a comparison of ACI with mosaicplasty was made by Bentley et al. the first in 2003 with a mean follow-up of 1.7 years and the second in 2012 with a minimum follow-up of 10 years [36, 37]. At the first follow-up, 9 of 42 mosaicplasty patients (21%) exhibited an excellent result in contrast to 23 out of 58 (40%) in the ACI group. Furthermore, the rate of poor results for the mosaicplasty patients was distinctly higher (17%) than in the ACI group (0%).

Arthroscopy at 1-year postoperatively demonstrated excellent or good repairs in 82% after ACI. Following mosaicplasty, 34% had good results, no “excellent” outcome. At a minimum of 10 years follow-up, the repair had failed in 10 out of 58 ACI patients (17%) and 23 out of 42 (55%) from the mosaicplasty group.

In this light, the main characteristics of an optimal surgical treatment for unstable OCD were the presence of an arthroscopic technique and procedure aiming at recreating a hyaline-like tissue to restore the chondral elements of the articular surface as similar as possible to the physiological one.

But the requirement of two surgical procedures and the high costs associated with cell expansion have been major drawbacks of ACI. Moreover, there is a frequent eventuality of periosteal hypertrophy and delamination, which often requires revision surgery. Technical problems were also identified, such as subsistence of chondrocyte phenotype, non-homogeneous cell distribution and cell loss using liquid suspension [38].

The rationale for the BMDC transplant with “one-step technique” is to transfer the entire regenerative niche without using the laboratory phase [21]. This allows cells to be handled directly in the operating room, without the requirement of a laboratory phase, and allows BMDC transplantation to be performed in “one step” instead of the two required for ACI [21, 39–41]. Aim and benefits of transplantation niche are to maintain the reciprocal regulatory interaction between the stem cells and the microenvironment, and also to transfer into the lesion site the entire regenerative potential present in the bone marrow mononuclear component [43, 44].

The results obtained in this study with the one-step technique in OCD repair closely be alike those obtained with other reparative techniques in similar lesions.

In our case series, a significant improvement both in IKDC, KOOS and TEGNER scores, from pre-op to each follow-up, was found (*p* < 0.05).

MRI examination with MOCART score showed satisfactory growth of bone and cartilage, nearly complete defect filling and good integration of the graft at follow-up in 80% of cases. Among various MRI parameters, only signal intensity was significantly correlated with the KOOS and IKDC score at especially at last follow-up.

In four patients, subchondral oedema was also present: Alparslan et al. reported that the presence of subchondral oedema had an influence on the clinical results; nevertheless, in our case series, this occurrence had no negative impact on clinical scores [42].

The limitations of this study include the small sample size of our case series. Our data are gathered in one institution only, and this may affect the external validity of the results. Although commonly used, the IKDC, KOOS and TEGNER scores are not validated for JOCD. The adult forms of the questionnaires were used. However, most of the patients had turned 18 when they answered these. In addition, our study lacked a control group. Finally, although the data have been collected prospectively, it is basically a retrospective study with possible unchecked biases.

## Conclusion

According to our results “One-step” technique, among regenerative osteochondral procedures, transferring the whole bone marrow aspirate niche permit articular surface restoration with viable physiologic osteochondral tissue providing clinical efficaciousness as demonstrated through MRI.

The number of cases is still limited and further studies with larger sample sizes and greater follow-up evaluations are required to confirm our results. Nevertheless, we believe that BMDCT may represent a suitable option to treat OCD lesion in young adults.

## References

[1] König F (1888). Ueber freie Körper in den Gelenken. Dtsch Z Chir.

[2] König F (2013). The classic: on loose bodies in the joint 1887. Clin Orthop Relat Res.

[3] Tarabella V, Filardo G, Di Matteo B, Andriolo L, Tomba P, Viganò A (2016). From loose body to osteochondritis dissecans: a historical account of disease d definition. Joints.

[4] Shea KG, Jacobs JC, Carey JL, Anderson AF, Oxford JT (2013). Osteochondritis dissecans knee histology studies have variable findings and theories of aetiology. Clin Orthop Relat Res.

[5] Kocher MS, Tucker R, Ganley TJ, Flynn JM (2006). Management of osteochondritis dissecans of the knee: current concepts review. Am J Sports Med.

[6] Chambers HG, Shea KG, Carey JL (2011). AAOS Clinical practice guideline: diagnosis and treatment of osteochondritis dissecans. J Am Acad Orthop Surg.

[7] Linden B (1976). The incidence of osteochondritis dissecans in the condyles of the femur. Acta Orthop Scand.

[8] Cooper T, Boyles A, Samora WP, Klingele KE (2015) Prevalence of Bilateral JOCD of the Knee and Associated Risk Factors. J Pediatr Orthop 35(5):507–10. 10.1097/bpo.0000000000000323. 10.1097/BPO.0000000000000323 25290254

[9] Cahill BR (1995). Osteochondritis dissecans of the knee: treatment of juvenile and adult forms. J Am Acad Orthop Surg.

[10] DeSmet AA, Ilahi OA, Graf BK (1996). Reassessment of the MR criteria for stability of osteochondritis dissecans in the knee and ankle. Skelet Radiol.

[11] Glancy GL (1999). Juvenile osteochondritis dissecans. Am J Knee Surg.

[12] Cahill BR, Phillips MR, Navarro R (1989). The results of conservative management of juvenile osteochondritis dissecans using joint scintigraphy: a prospective study. Am J Sports Med.

[13] Wright RW, McLean M, Matava MJ, Shivelt RA (2004). Osteochondritis dissecans of the knee: long-term results of excision of the fragment. Clin Orthop Relat Res.

[14] Yonetani Y, Tanaka Y, Shiozaki Y, Kanamoto T, Kusano M, Tsujii A, Horibe S (2012) Transarticular drilling for stable juvenile osteochondritis dissecans of the medial femoral condyle. Knee Surg Sports Traumatol Arthrosc 20(8):1528-32.10.1007/s00167-011-1736-1. 10.1007/s00167-011-1736-1 22072323

[15] Kocher MS, Micheli LJ, Yaniv M, Zurakowski D, Ames A, Adrignolo AA (2001). Functional and radiographic outcome of juvenile osteochondritis dissecans of the knee treated with transarticular arthroscopic drilling. Am J Sports Med.

[16] Camp CL, Krych AJ, Stuart MJ (2013). Arthroscopic preparation and internal fixation of an unstable osteochondritis dissecans lesion of the knee. Arthrosc Technol.

[17] Steadman JR, Briggs KK, Rodrigo JJ, Gill TJ (2003). Outcomes of patients treated arthroscopically by microfracture for traumatic chondral defects of the knee: average 11-year follow-up. Arthroscopy.

[18] Gudas R, Simonaityte R, Cekanauskas E, Tamosiunas R (2009). A prospective, randomized clinical study of osteochondral autologous transplantation versus microfracture for the treatment of osteochondritis dissecans in the knee joint in children. J Pediatr Orthop.

[19] Peterson L, Minas T, Brittberg M, Lindahl A (2003). Treatment of osteochondritis dissecans of the knee with autologous chondrocyte transplantation: results at two to ten years. J Bone Joint Surg Am.

[20] Filardo G, Kon E, Di Martino A (2013). Treatment of knee osteochondritis dissecans with a cell-free biomimetic osteochondral scaffold: clinical and imaging evaluation at 2-year follow-up. Am J Sports Med.

[21] Giannini S, Buda R, Vannini F, Cavallo M, Grigolo B (2009). One-step bone marrow-derived cell transplantation in talar osteochondral lesions. Clin Orthop Relat Res.

[22] Daniele N, Scerpa MC, Rossi C, Lanti A, Adorno G, Isacchi G, Zinno F (2014) The processing of stem cell concentrates from the bone marrow in ABO-incompatible transplants: how and when. Blood Transfus 12:150–158. 10.2450/2013.0127-13 10.2450/2013.0127-13 PMC4039695 24333081

[23] Song F, Tang J, Geng R, Hu H, Zhu C, Cui W, Fan W (2014). Comparison of the efficacy of bone marrow mononuclear cells and bone mesenchymal stem cells in the treatment of osteoarthritis in a sheep model. Int J Clin Exp Pathol.

[24] Buda R, Vannini F, Cavallo M, Baldassarri M, Luciani D, Mazzotti A, Pungetti C, Olivieri A, Giannini S (2013). One-step arthroscopic technique for the treatment of osteochondral lesions of the knee with bone-marrow-derived cells: three years results. Musculoskelet Surg.

[25] Salter RB (1994). The physiological basis of continuous passive motion for articular cartilage healing and regeneration. Hand Clin.

[26] Detterline AJ, Goldstein JL, Rue JP, Bach BR (2008). Evaluation and treatment of osteochondritis dissecans lesions of the knee. J Knee Surg.

[27] Ramirez A, Abril JC, Chaparro M (2010). Juvenile osteochondritis dissecans of the knee: perifocal sclerotic rim as a prognostic factor of healing. J Pediatr Orthop.

[28] Capone C, Frigerio S, Fumagalli S et al (2007) Neurosphere-derived cells exert a neuroprotective action by changing the ischemic microenvironment. PLoS One 2:e373 10.1371/journal.pone.0000373 PMC1847533 17440609

[29] Clanton TO, DeLee JC (1982). Osteochondritis dissecans: history, pathophysiology and current treatment concepts. Clin Orthop Relat Res.

[30] Andriolo L, Crawford DC, Reale D, Zaffagnini S, Candrian C, Cavicchioli A, Filardo G (2018) Osteochondritis dissecans of the knee: etiology and pathogenetic mechanisms. a systematic review. Cartilage. 10.1177/1947603518786557 10.1177/1947603518786557 PMC7298596 29998741

[31] Tarabella V, Filardo G, Di Matteo B (2016). From loose body to osteochondritis dissecans: a historical account of disease definition. Joints.

[32] Brittberg M, Lindahl A, Nilsson A (1994). Treatment of deep cartilage defects in the knee with autologous chondrocyte transplantation. N Engl J Med.

[33] Cole BJ, DeBerardino T, Brewster R (2012). Outcomes of autologous chondrocyte implantation in Study of the Treatment of Articular Repair (STAR) patients with osteochondritis dissecans. Am J Sports Med.

[34] Moriya T, Wada Y, Watanabe A (2007). Evaluation of reparative cartilage after autologous chondrocyte implantation for osteochondritis dissecans: histology, biochemistry, and MR imaging. J Orthop Sci.

[35] Ferruzzi A, Buda R, Faldini C, Vannini F, Di Caprio F, Luciani D (2008). Autologous chondrocyte implantation in the knee joint: open compared with arthroscopic technique. J Bone Joint Surg Am.

[36] Bentley G, Biant LC, Carrington RW, Akmal M, Goldberg A, Williams AM (2003). A prospective, randomised comparison of autologous chondrocyte implantation versus mosaicplasty for osteochondral defects in the knee. J Bone Joint Surg Br.

[37] Bentley G, Biant LC, Vijayan S, Macmull S, Skinner JA, Carrington RW (2012). Minimum ten-year results of a prospective randomised study of autologous chondrocyte implantation versus mosaicplasty for symptomatic articular cartilage lesions of the knee. J Bone Joint Surg Br.

[38] Niethammer TR, Pietschmann MF, Horng A, Rossbach BP, Ficklscherer A, Jansson V (2014). Graft hypertrophy of matrix-based autologous chondrocyte implantation: a two-year follow-up study of NOVOCART 3D implantation in the knee. Knee Surg Sports Traumatol Arthrosc.

[39] Vannini F, Battaglia M, Buda R, Cavallo M, Giannini S (2012). “One step” treatment of juvenile osteochondritis dissecans in the knee: clinical results and T2 mapping characterization. Orthop Clin North Am.

[40] Taichman RS (2005). Blood and bone: two tissues whose fates are intertwined to create the hematopoietic stem-cell niche. Blood.

[41] Lepore AC, Han SS, Tiler-Polsz CJ, Cai J, Rao MS, Fischer I (2004). Transplantation into the adult CNS. Neuron Glia Biol.

[42] Alparslan L, Winalski CS, Boutin RD (2001). Postoperative magnetic resonance imaging of articular cartilage repair. Semin Musculoskelet Radiol.

[43] Olmsted-Davis EA, Gugala Z, Camargo F, Gannon FH, Jackson K, Kienstra KA, Shine HD, Lindsey RW, Hirschi KK, Goodell MA, Brenner MK, Davis AR (2003). Primitive adult hematopoietic stem cells can function as osteoblast precursors. Proc Natl Acad Sci U S A.

[44] Caplan AI (2009). Why are MSCs therapeutic?. New data: new insight J Pathol.

