# Correction to: Osteocondritis dissecans lesions of the knee restored by bone marrow aspirate concentrate: clinical and imaging results in 18 patients

**DOI:** 10.1007/s00590-023-03655-2

**Published:** 2023-08-03

**Authors:** Matteo Baldassarri, Roberto Buda, Luca Perazzo, Diego Ghinelli, Ricciardello Sarino, Brunella Grigolo, Cesare Faldini

**Affiliations:** 1 Villa Maria Hospital, Viale Matteotti, 4, 47921 Rimini, Italy; 2 Orthopaedic and Traumatology Clinic, Ospedale “SS. Annunziata” - Università Degli Studi Gabriele d’Annunzio - Chieti e Pescara, Via Dei Vestini, 31, 66100 Chieti, Italy; 3 Casa Di Cura Villa Laura, Via Emilia Levante, 137, 40139 Bologna, Italy; 4 RAMSES Laboratory, IRCCS Istituto Ortopedico Rizzoli, Via Di Barbiano 1/10, 40136 Bologna, Italy; 5 I Orthopaedic and Traumatology Clinic, IRCCS Istituto Ortopedico Rizzoli – Bologna, Via G. C. Pupilli, 1, 40136 Bologna, Italy; 6 UNIZKM: Università Cattolica Nostra Signora del Buon Consiglio, Tirana – Albania, Via A. Poliziano, 2, 40129 Bologna (BO), Italy

**Correction to: European Journal of Orthopaedic Surgery & Traumatology (2023) 33:857–867** 10.1007/s00590-022-03214-1

The original version of this article unfortunately contained a mistake. The affiliation details for Authors Brunella Grigolo and Cesare Faldini were incorrectly given.

The correct affiliations are given below.

Brunella Grigolo

RAMSES Laboratory, IRCCS Istituto Ortopedico Rizzoli, Via di Barbiano 1/10—40,136, Bologna, Italy.

Cesare Faldini

I Orthopaedic and Traumatology Clinic, IRCCS Istituto Ortopedico Rizzoli – Bologna, Via G. C. Pupilli, 1 – 40,136 Bologna, Italy.

